# Meteorological Register for July, 1816

**Published:** 1816-09

**Authors:** John Bailey

**Affiliations:** Surgeon, &c.


### METEOROLOGICAL REGISTER for JULY, 1816.

*Kept at Harwich, in Essex.*

By JOHN BAILEY, Surgeon, &c.

| | Moon's Age. | Atmospherical Pressure. Morn. | Ev. | Temp. | Wind. | Remarks. | General Statement of Diseases. |
|---|---|---|---|---|---|---|---|
| | 1 | 29 6½ | 29 7 | 60 | W | Rain | Rheumatismus |
| | 2 | 7 | 7 | 66 | W | | acutus |
| ☾ | 3 | 7 | 7½ | 68 | W | ditto | |
| | 4 | 7 | 7 | 66 | SW | Heavy showers of rain | Pertussis |
| | 5 | 6 | 7½ | 62 | NW | ditto | |
| | 6 | 7½ | 7½ | 64 | W | Rain & very dull | Ophthalmia |
| | 7 | 6½ | 6 | 64 | SW | Rain | |
| | 8 | 6 | 6 | 68 | SW | ditto | Intermittens |
| ○ | 9 | 5 | 5 | 66 | W | ditto | |
| | 10 | 5 | 5½ | 68 | SW | ditto | Scarlatina |
| | 11 | 6½ | 7½ | 65 | NW | ditto | |
| | 12 | 8 | 8 | 65 | N | ditto | Varicella |
| | 13 | 8 | 6½ | 64 | SW | ditto | |
| | 14 | 5½ | 6 | 68 | W | ditto | Pneumonia |
| | 15 | 6 | 5 | 65 | SW | Heavy showers of rain | |
| | 16 | 5 | 5 | 64 | W | Rain | |
| ☽ | 17 | 5½ | 3½ | 64 | SW | Ditto. G. of w. | |
| | 18 | 3 | 5 | 65 | SW | ditto | |
| | 19 | 5 | 6 | 58 | S | Heavy rain | |
| | 20 | 6½ | 6 | 69 | E | Brisk wind | |
| | 21 | 5 | 6 | 71 | W | Rain. B. wind | |
| | 22 | 7 | 7 | 67 | W | Rain | |
| | 23 | 6½ | 5½ | 65 | SW | ditto | |
| ● | 24 | 5½ | 5 | 66 | W | ditto | |
| | 25 | 5 | 6½ | 68 | NW | ditto | |
| | 26 | 7 | 8 | 68 | NW | ditto | |
| | 27 | 8 | 8 | 65 | NW | ditto | |
| | 28 | 7 | 7 | 64 | NW | ditto | |
| | 29 | 6½ | 5 | 67 | E | | |
| | 30 | 5 | 4½ | 64 | Var. | Rain | |
| ☾ | 31 | 4 | 4 | 65 | NW | Do. Very dull | |

The almost incessant rain with which the soil in this neighbourhood has been covered, has given rise to the presence of intermittents.—Rheumatism, of the acute kind, and pulmonary attacks have likewise been pretty general.—Scarlatina does not spread with activity, although a moist state of the atmosphere, which is supposed favourable to its propagation, exists. It seems that a low temperature of the air must co-exist, to give much activity to the contagion of this disease.—Pertussis and Ophthalmia are not general.

